# Gene Targets in Ocular Pathogenic *Escherichia coli* for Mitigation of Biofilm Formation to Overcome Antibiotic Resistance

**DOI:** 10.3389/fmicb.2019.01308

**Published:** 2019-06-21

**Authors:** Konduri Ranjith, Jahnabi Ramchiary, Jogadhenu S. S. Prakash, Kotakonda Arunasri, Savitri Sharma, Sisinthy Shivaji

**Affiliations:** ^1^Jhaveri Microbiology Centre – Prof. Brien Holden Eye Research Centre, LV Prasad Eye Institute, Hyderabad, India; ^2^Research Scholar, Manipal Academy of Higher Education, Manipal, India; ^3^Department of Biotechnology and Bioinformatics, School of Life Sciences, University of Hyderabad, Hyderabad, India

**Keywords:** antimicrobial resistance, biofilm, ocular, *E. coli*, endophthalmitis, mutation

## Abstract

The present work is an attempt to establish the functionality of genes involved in biofilm formation and antibiotic resistance in an ocular strain of *Escherichia coli* (L-1216/2010) which was isolated and characterized from the Vitreous fluid of a patient with Endophthalmitis. For this purpose, seven separate gene-specific knockout mutants were generated by homologous recombination in ocular *E. coli*. The genes that were mutated included three transmembrane genes *ytfR* (ABC transporter ATP-binding protein), *mdtO* (multidrug efflux system) and *tolA* (inner membrane protein), *ryfA* coding for non-coding RNA and three metabolic genes *mhpA* (3-3-hydroxyphenylpropionate 1,2-dioxygenase), *mhpB* (2,3-di hydroxyphenylpropionate 1,2-dioxygenase), and *bdcR* (regulatory gene of *bdcA*). Mutants were validated by sequencing and Reverse transcription-PCR and monitored for biofilm formation by XTT method and confocal microscopy. The antibiotic susceptibility of the mutants was also ascertained. The results indicated that biofilm formation was inhibited in five mutants (Δ*bdcR*, Δ*mhpA*, Δ*mhpB*, Δ*ryfA*, and Δ*tolA*) and the thickness of biofilm reduced from 17.2 μm in the wildtype to 1.5 to 4.8 μm in the mutants. Mutants Δ*ytfR* and Δ*mdtO* retained the potential to form biofilm. Complementation of the mutants with the wild type gene restored biofilm formation potential in all mutants except in Δ*mhpB*. The 5 mutants which lost their ability to form biofilm (Δ*bdcR*, Δ*mhpA*, Δ*mhpB*, Δ*tolA*, and Δ*ryfA*) did not exhibit any change in their susceptibility to Ceftazidime, Cefuroxime, Ciprofloxacin, Gentamicin, Cefotaxime, Sulfamethoxazole, Imipenem, Erythromycin, and Streptomycin in the planktonic phase compared to wild type ocular *E. coli*. But Δ*mdtO* was the only mutant with altered MIC to Sulfamethoxazole, Imipenem, Erythromycin, and Streptomycin both in the planktonic and biofilm phase. This is the first report demonstrating the involvement of the metabolic genes *mhpA* and *mhpB* and *bdcR* (regulatory gene of *bdcA*) in biofilm formation in ocular *E. coli*. In addition we provide evidence that *tolA* and *ryfA* are required for biofilm formation while *ytfR* and *mdtO* are not required. Mitigation of biofilm formation to overcome antibiotic resistance could be achieved by targeting the genes *bdcR, mhpA, mhpB, ryfA*, and *tolA*.

## Introduction

Antimicrobial resistance (AMR) refers to the ability of microorganisms (bacteria, fungi, viruses, and parasites) to resist the inhibitory effect of an antimicrobial agent at the minimum inhibitory concentration (MIC) of the antimicrobial agent. AMR is a global phenomenon and bacteria, fungi and viruses are known to exhibit AMR. Various factors intrinsic to the microbe are also known to facilitate AMR such as their ability to inactivate the antimicrobial agent, to prevent its entry, to activate its efflux, to modify the antibiotic or the target, acquire resistance genes, and the ability to form a biofilm. Over the years with the development of the crystal violet method, XTT method, confocal microscopy and scanning electron microscopy it has been possible to monitor the dynamics of biofilm formation, and evaluate simultaneously the extent of AMR. These studies have clearly established that biofilm confers AMR to microorganisms (Hoyle et al., 1992; Elder et al., 1995; Ranjith et al., 2017). Several ocular pathogens like *Pseudomonas aeruginosa, Staphylococcus aureus, S. epidermidis, Micrococcus luteus, Serratia marcescens, Neisseria* spp., *Moraxella* spp., *Bacillus* spp., *E. coli, Proteus mirabilis, Enterobacter agglomerans*, and *Klebsiella* spp., also exhibited the potential to form biofilms and were resistant to antibiotics (Katiyar et al., 2012). Recently we demonstrated that ocular *E. coli* from patients with conjunctivitis, keratitis or endopthalmitis were resistant to one or more of the antibiotics tested and majority of the isolates were positive for biofilm formation. *E. coli* L-1216/2010, was the best biofilm forming ocular isolate, and in the biofilm phase it was 100 times more resistant to the 8 antibiotics tested compared to planktonic phase (Ranjith et al., 2017). In the same study it was also demonstrated by DNA microarray analysis that ocular *E. coli* L-1216/2010 in the biofilm phase over-expressed several genes (Ranjith et al., 2017). But, comparison of the expression pattern of specific genes in ocular *E. coli* L-1216/2010 and in pathogenic *E. coli* ABU strain 83972 (Hancock and Klemm, 2007) and in *E. coli* UPEC strain CFT073 (Schembri et al., 2003) were distinctly different though both are pathogens. For instance, *fim* genes coding for type 1 fimbriae, genes coding for the LPS lipid A moiety (*waaB, waaP, waaJ*, and *waaR*), *yhjN* coding for cellulose synthase regulator protein, were up regulated in ocular *E. coli* L-1216/2010 but not in *E. coli* ABU strain 83972 (Hancock and Klemm, 2007) and in *E. coli* UPEC strain CFT073 (Schembri et al., 2003). Further the 22 genes (c2418 to c2440) that constitute the pathogenicity island and *rfaH* a virulence regulator, which regulates expression of several virulent genes in *E. coli* were up regulated during biofilm formation in several pathogenic strains but not in ocular *E. coli* L-1216/2010 (Schembri et al., 2003; Ranjith et al., 2017). It was also observed that ocular *E. coli* in biofilm phase did not exhibit up regulation of the stress response encoding genes (such as *cspG, cspH, pphA, ibpA, ibp, soxS, hha*, and *yfiD*) as observed in ABU strains of *E. coli*. These results indicate differences between the ocular and the non-ocular pathogenic *E. coli* in expression of genes in the biofilm phase.

In the present study a few genes viz., *mhpA* [coding for 3-(3-hydroxyphenyl) propanoate hydroxylase], *mhpB* (coding *for* 2,3-dihydroxyphenylpropionate 1,2-), *ryfA* (coding for non-coding RNA), *tolA* (coding for the inner membrane protein -cell envelope integrity), *bdcR* (coding for the regulatory gene of *bdcA*), *ytfR* (coding for ABC transporter ATP-binding protein), and *mdtO* (coding for multidrug efflux system component) known to be up regulated in expression during biofilm formation in ocular *E. coli* L-1216/2010 (Ranjith et al., 2017) were mutated by homologous recombination based on the procedure of Datsenko and Wanner (2000) to validate the function of the above genes in biofilm formation in ocular pathogenic *E. coli*. The choice of the seven genes for gene specific mutation was based on the following criteria:

(a) genes known to be required for biofilm formation like *ryfA* (Bak et al., 2015) in *E. coli* and *tolA* in *P. aeruginosa* (Whiteley et al., 2001) and *E. coli* (Vianney et al., 2005) and (b) genes up regulated in expression during biofilm phase like *bdcR, mdtO, mhpA, mhpB*, and *ytfR* (Ranjith et al., 2017) but whose specific role in biofilm formation has not yet been demonstrated.

Such studies would help to identify genes involved in biofilm formation in ocular *E. coli* directly, establish whether genes known to be involved in biofilm formation in other organisms have a similar function in ocular *E. coli* and may also establish if there is a relationship between biofilm formation and resistance to antimicrobial agents and vice a versa.

## Materials and Methods

### Bacterial Strains and Plasmids

Ocular *E. coli* L-1216/2010 which was isolated and characterized from the Vitreous fluid of a patient with Endophthalmitis and resistant to ciprofloxacin and positive for biofilm formation was used in the present study (Ranjith et al., 2017). The bacterium was cultured in LB medium at 37°C. Several mutant strains of this bacterium (Table 1) were generated by homologous recombination (see below). Growth was monitored spectrophotometrically at 600 nm for 24 h.

**Table 1:** Bacterial strains of *E. coli* L-1216/2010, plasmids and PCR primers used in the study.

Plasmids and isolates used in this study			
pKD3		Amp^r^, Chlrm^r^	CDFD^∗^
pKD46		Amp^r^	CDFD^∗^
pET28a		Amp^r^	UoH^#^
*E. coli* DH5α			LVPEI^$^
Ocular *E. coli*		Biofilm forming ocular *E. coli* isolate L-1216/2010	This study

**Primers for generating mutants^∗^**			Amplicon size (bp)

Δ*mhpA*		F-ATGGCAATACAACACCCTGACATCCAGCCTGCGTGTAGGCTGGA GCTGCTTCGAAGTTCC R- TCAGGCTACCTTTTCGACAGAAACGTCGGCATCAGGCATATGAAT ATCCTCCTTAGTTCC	1082
Δ*mhpB*		F- ATGCACGCTTATCTTCACTGTCTTTCCCACTCGCCGGTGTAGGCT GGAGCTGCTTCGAAGTTCC R- TCAGTTCTCTGTTCTGGCGCTTAACGAGCCAAATCCCATATGAAT ATCCTCCTTAGTTCC	1086
Δ*bdcR*		F- ATGGTCACTAAAAAACAATCTCGCGTTCCAGGTCGTCCCGTGTA GGCTGGAGCTGCTTCGAAGTTCC R- TCACTCCTCAAGAATAGTTTTTATCGCTTCCCCCGCCATATGAAT ATCCTCCTTAGTTCC	1089
Δ*tolA*		F- GTGTCAAAGGCAACCGAACAAAACGACAAGCTCAAGCGGGTGT AGGCTGGAGCTGCTTCGAAGTTCC R- TTACGGTTTGAAGTCCAATGGCGCGTTTTTGAACACCATATGAAT ATCCTCCTTAGTTCC	1089
Δ*rfyA*		F- GCGGCCCTTTCCGCCGTCTCGCAAACGGGCGCGTGTAGGCTGGA GCTGCTTCGAAGTTCC R- AAAATATGCCGCCCAAAGGGCGGCATTCGGGCCATATGAATATC CTCCTTAGTTCC	1078
Δ*mdtO*		F- ATGAGCGCGCTCAACTCCCTGCCATTACCGGTGGTCGTGTAGGC TGGAGCTGCTTCGAAGTTCC R-TCATTGCGTGGCTCCTTGTGCCTGTTCCGTGGCGGGCATATGAAT ATCCTCCTTAGTTCC	1086
Δ*ytfR*		F- ATGACGACCGACCAACACCAGGAGATCCTCCGCACCGTGTAGGC TGGAGCTGCTTCGAAGTTCC R- TTACGCCGCAATGGCGTTCATGATCGCCGGAACGGACATATGAA TATCCTCCTTAGTTCC	1086

**Primers for complementation**			Amplicon size

p*mhpA-F*		F- AAGCTT ATGGCAATACAACACCCTGACATCC R-ACGGAGCTTATTTGTTGTTGCGCAGATCCAGC	6675
p*mhpB-F*		F- AAGCTT ATGCACGCTTATCTTCACTGTCTTTCC R-ACGGAGCTTATTTGTTGTTGCGCAGATCCAGC	5465
p*bdcR*		F-AGCCATATGGTCACTAAAAAACAATCTCG R-ACGGAGCTCTCACTCCTCAAGAATAGTTTTTATCG	605
p*tolA*		F-AGCCATGTGTCAAAGGCAACCGAACAAAACG R-ACGGAGCTCTTACGGTTTGAAGTCCAATGGCGCG	1276
p*rfyA*		F-AGCCATGCGGCCCTTTCCGCCGTCTCGCAAACG R-ACGGAGCTCAAAATATGCCGCCCAAAGGGCGGC	315

**Primers for Reverse transcription-PCR (validation of mutants)**			Amplicon size

Rt-*mhpA*	F- TGGCGATCGCTGGTGCCGGCCCGG R- CGATCAACTTATCGAGTTTCTCC		126
Rt-*mhpB*	F- GGTATGTCGACCCGGCGCAAGAGG R- AAAAGCCGTTGTAGTGATCTGG		135
Rt-*bdcR*	F- CCCAGACGTTTCGCTCCTGAGCAGGC R- TTAATACCAAGATAATCAGTAAC		98
Rt-*tolA*	F- GTGCTGCATGTCATCTTATTTGCGG R- CCGCACCTGAATCAACCATGACAGCG		116
Rt-*rfyA*	F- AGGAAAGGATGTTCCGTGGCCG R- TCTTCATTGTGCGGTCCATGC		132
Rt-*mdtO*	F- ATCCCTTTTGTGGCGTTATCACTGG R- AACAGGCTGGCGATTTCCAGC		106
Rt-*ytfR*	F- CCGGCGTCAAAGCGTTAGACAACG R- GATGGTGCCGCGATCGGCGTGG		119
**Primers for sequencing (validation of mutants)**			

Seq-*mhpA*	F- TTCCGGCCACCAGAATAGCC (-200 bp from ATG of *mhpA*) R- TGCTTAATGAATACAACAGTACTGCG		375
Seq-*mhpB*	F- GCCATTCCGCAAACCCTGGGCAAGACCC (-200 region from ATG of *mhpB* gene) R- TGCTTAATGAATACAACAGTACTGCG		375
Seq-*bdcR*	F-CGTACGATAGCGGCACCGATACC (-198 bp from ATG of *bdcR*) R- TGCTTAATGAATACAACAGTACTGCG		373
Seq-*tolA*	F- ACCCGAAAACGGTCTTTCTGATCGG (-180bp from GTG of *tolA*) R- TGCTTAATGAATACAACAGTACTGCG		353
Seq-*rfyA*	F-GGCGGTTTTTTGTTGCTGGTCCGG (-176 bp from GCG of *ryfA*) R- TGCTTAATGAATACAACAGTACTGCG		349
Seq-*mdtO*	F- GAGGGTAAAGTGGATTCGATTGGC (-189 from ATG of *mdtO*gene) R- TGCTTAATGAATACAACAGTACTGCG		364
Seq-*ytfR*	F-TCCGGCGATGGTGCACTGAAGCC (-186 from ATG of *ytfR* gene) R- TGCTTAATGAATACAACAGTACTGCG		361

*All the hybrid primers in this group contain 36 nucleotides of the target gene (either downstream or upstream sequence depending on whether it is a forward or reverse primer) plus primer for the antibiotic cassette such that the PCR amplicon would contain 36 nucleotides of the target gene from either end plus the antibiotic cassette gene. ^∗^CDFD, center for DNA fingerprinting and diagnostics. ^#^UoH, University of Hyderabad. ^$^LVPEI-L V Prasad Eye Institute.*

### Biofilm Detection by XTT Method

Culture (200 μl of 1:10 dilution of 0.5 McF culture) was added to a 96 well plate and incubated for 72 h at 37°C. XTT [2,3-Bis-(2-Methoxy-4-Nitro-5-Sulfophenyl)-2*H*-Tetrazolium-5-Carboxanilide] (50 μl of 1 mg/ml) (Sigma, United States) and 4.2 μl (0.5 mM) Menadione and 145.8 μl of 1X PBS was added to each well and incubated in the dark for 3 h and quantified using a spectrophotometer (SpectroMax M3, Molecular Devices, CA, United States) at 490 nm. Wells without culture served as the control and the control OD at 490 nm was <0.12.

### Visualization of Biofilm by Confocal Laser Scanning Microscopy (CLSM)

*Escherichia coli* L-1216/2010 and the mutants were cultured on μ- chamber slide 8 well (ibidi, Gmbh, Germany) for 72 h as above and the biofilms were rinsed twice gently with autoclaved water and fixed with 4% formaldehyde (Himedia-Secunderabad, India) for 45 min. Fixed biofilms were then washed twice as above and stained with 200 μl of 1.67 μM Syto^®^9 a nuclear fluorescent dye (Invitrogen, United States) for 30 min. Stained biofilms were washed again with autoclaved water. Confocal images were taken using Zeiss confocal laser scanning microscope (Carl Zeiss LSM 880, Jena, Germany). Argon Laser was excited at 450–490 nm and a 40× objective was used set at Zoom 2.

### EPS Production Using Calcofluor White Staining

After staining of the cells in the biofilm with Syto 9, the biofilms were stained in the dark with 0.025% Calcofluor white M2R (Sigma Chemical Co., St. Louis, MO, United States) for 30 min. This dye binds to β-linked polysaccharides and fluoresces under long-wave UV light (Wood, 1980) and biofilm could be visualized (blue) using confocal microscopy. Calcofluor white has been used to study exopolysaccharides (EPSs) involved in biofilm formation in a variety of organisms (Zogaj et al., 2001; Ledeboer and Jones, 2005). Calcofluor white was excited at 363-nm using a 455/30 band-pass filter (Cowan et al., 2000).

### Production of Curli and Cellulose Nanofibers by *E. coli* Strains by the Congo Red-Binding Assay

Curli and cellulose nanofibers production in *E. coli* L-1216/2010 and the mutants was monitored by the Congo red-binding assay method (Arita-Morioka et al., 2015) using Congo red-containing YESCA agar plates. *E. coli* L-1216/2010 and the mutants were cultured overnight and then 10 μl of the culture were spotted on YESCA media plates containing 10 g/l casamino acids, 10 g/ml Coomassie brilliant blue G-250, 1 g/l yeast extract, and 20 g/L agar. The plates were incubated at 30°C for 3 days and the colonies were photographed. Colonies positive for curli and cellulose nanofibers production were red and colonies positive only for cellulose nanofibers were pink in color and could be easily differentiated.

### Quantification of the Attachment of Cells to the Substratum by SYTO9 Staining

Adhesion of cells to the substratum was assessed using the procedure of Schmidt-Emrich et al. (2016). Overnight culture was adjusted to 0.5 McFarland units, diluted 100-fold with the medium and 100 μl of the diluted inoculum was dispensed into a single well of a 96 well plate (Thermo Fisher Scientific, Nunclon^TM^, Denmark) containing 100 μl of fresh medium. The plate was incubated at 37 °C for 4 h. The broth was then discarded by inverting the plate and gently tapping it after which it was washed thrice with 200 μl of phosphate buffered saline (PBS, Sigma-Aldrich Corporation, St. Louis, MO, United States) and allowed to dry for 30 min. The bacteria in the biofilm adhering to the plate were then stained with 200 μl of 2.5 μM SYTO9 in dark and incubated for 15 min at room temperature on a shaker and quantified using a spectrophotometer (SpectroMax M3, Molecular Devices, CA, United States) at 490 nm. Wells without culture served as the control.

### Motility of Ocular *E. coli* L-1216/2010 and the Mutants by Monitoring Swimming and Swarming of Cells

Swimming motility is an individual and random-directional movement in liquid medium, and essential for initial attachment to develop biofilm. Swarming motility is a coordinated bacterial social movement across the top of a solid surface, accompanied by hyper-flagellation critical for surface colonization after initial attachment. Swimming and swarming motilities were investigated (Bak et al., 2015) on specific agar plates as indicated below. For this purpose 1 μl of 0.5 mcF (∼5 × 10^4^ cells) of each mutant was inoculated with a pipette tip onto swim agar (0.3% Bacto Agar, 1% tryptone and 0.5% NaCl) or swarm agar (0.6% Eiken Agar, 0.5% glucose, 1% tryptone, 0.5% yeast extract and 0.5% NaCl). The swimming assay was performed at 30 °C for 12 h, and swarming assay at 37 °C for 16 h, respectively. The diameter of the swimming circle or the diameter of swarming area was measured and normalized against that of a wild type strain (Bak et al., 2015).

### Antibiotic Susceptibility of Ocular *E. coli* L-1216/2010 and the Mutants

The minimum inhibitory concentration (MIC) of the antibiotic was determined by the micro-dilution method as described by the European Committee on Antimicrobial susceptibility Testing (CLSI, 2012). Antibiotics were obtained from commercial sources and each concentration was tested thrice. The susceptibility of the strains was also determined after the formation of the biofilm. For this purpose, the cultures were incubated as above in the 96 well plate at 37°C for 48 h for biofilm formation. Planktonic cells were discarded, bound cells washed with milliQ water and then the antibiotic dissolved in BHI medium (HiMedia, Mumbai, India) was added to the biofilm and incubated for 16 h. Biofilm formation was then monitored by the crystal violet method. The concentration of the antibiotic that inhibited the formation of the biofilm was determined. *E. coli* isolate L-1216/2010 in the planktonic phase (where in antibiotics were added after 24 h of growth) was used as controls for this experiment.

### Isolation of Plasmids, Transformation by CaCl_2_ Method, Preparation of Electro-Competent Cells, and Electroporation

Plasmids pKD3 and pKD46 were maintained in DH5α in LB medium with Ampicillin (100 μg/ml) (HiMedia, Mumbai, India) and the plasmids were isolated from an overnight 5 ml culture using Qiagen plasmid mini kit (Qiagen, Heilden, Germany). pKD46 plasmid containing the lambda red recombinase (plasmid gifted by CDFD, Hyderabad, India) gene was transformed into ocular *E. coli* by CaCl_2_ heat shock method (42°C for 45 s) and was plated on to LB agar plates supplemented with Ampicillin (100 μg/ml) and incubated at 30°C overnight. Colonies were then selected and grown in the same medium for future use.

Electro-competent cells of *E. coli* pKD46 were prepared using a standard protocol. An overnight culture diluted 1:100 in LB medium was inoculated into fresh LB medium and incubated at 30°C until the O.D_600_ reached 0.1.L-arabinose (10 mM, final concentration) was added to the culture and incubated further to OD_600_ of 0.6 to induce the expression of recombinase gene. The cells were then pelleted by centrifugation at 4000 rpm, washed thrice with autoclaved chilled MilliQ water, and finally resuspended in MilliQ water. The prepared electro-competent cells were stored at -80°C until use.

### Construction of Mutants in Biofilm Forming Ocular *E. coli* Isolate L-1216/2010

Mutant strains of *E. coli* were generated by homologous recombination based on the procedure of Datsenko and Wanner (2000). *E. coli* L-1216/2010 was first transformed by the CaCl_2_ heat shock method with pKD46 which expresses the Recombinase gene (*E. coli* pKD46) to facilitate homologous recombination). Subsequently, electro-competent cells of *E. coli* pKD46 were electroporated with a linear DNA fragment of the target gene using the Gene-Pulser-Xcellelectroporator (BioRad, Osaka, Japan), at 2.5 kV, 200 Ohm, and 25 μF. For this purpose the electro-competent ocular *E. coli* pKD46 cells were transferred to pre-chilled electroporation cuvettes (in ice for 10 min) and 5 μg of the PCR amplified linear DNA fragment was added and electroporated. LB medium (1 ml) (0.5% Yeast extract, 1% Peptone, 1% sodium chloride, and 1.5% Agar) was added immediately to the cells after applying the pulse and the electroporated cells were incubated at 37°C for 2 h and plated on an LB plate containing chloramphenicol (25 μg/ml) (HiMedia). Transformants were then selected on chloramphenicol plates and mutants of the target gene were validated by sequencing and Reverse transcription-PCR. Prior to electroporation the linear fragment was obtained by using hybrid primers (FH and RH). FH consisted of 36 nucleotides of the 5′ end of the gene of interest along with the forward primer of the cassette and RH contained 36 nucleotides of the 3′ end of the gene of interest along with the reverse primer of the cassette. When these primers were used they amplified a linear fragment from pKD3 which would contain 36 nucleotides of the target gene from either end plus the FRT (Flippase Recognition Target) region, the chloramphenicol resistance gene cassette and the FRT minimal region (1100 bp). This linear fragment was amplified (Table 1) for 35 cycles (each cycle: 94°C for 5 min, 94°C for 1 min, 63°C for 90 s, 72°C for 2 min, and finally 72°C for 15 min) using *pfu* polymerase enzyme (Thermo Fisher Scientific, MA, United States).

### Complementation of Mutants Using pET28a(+) Vector

#### (i) Amplification of Gene From Ocular *E. coli* Genome

For complementation studies the genes *bdcR, mhpA, mhpB, ryfA*, and *tolA* were amplified along with 200 bp upstream region using *pfu* polymerase (Table 1). Primers were designed based on the whole genome sequence of *E. coli* MG1655. Amplification was done for 35 PCR cycles as follows: 94°C for 5 min, 35× (94°C for 1 min, 56°C for 60 s, 72°C for 1.30 min), 72°C for 15 min. The PCR amplified products were then purified using Nucleospin Gel and PCR cleanup kit (Nucleospin, Duren, Germany) and then digested using Hind III/*NdeI* and XhoI/*SacI* restriction enzymes for 30 min at 37°C. These digested products would be ligated to pET28a(+) vector (as below).

#### (ii) Ligation of Gene Product From Dtep (i) With pET28a(+) Vector

Plasmid pET28a(+) (gift from J S SPrakash, University of Hyderabad, India) was isolated using plasmid isolation kit (Qiagen, Hilden, Germany) and 1 μg of pET28a(+) was digested with 1 μl of HindIII/*NdeI* and 1 μl of XhoI/*SacI* (NEB, Massachusetts, United Kingdom) for 30 min at 37°C. Later the digested vector was purified using Nucleospin Gel and PCR cleanup kit (Duren, Germany) and then ligated to *NdeI* and *SacI* digested target gene using T4DNA ligase (Thermo Fisher Scientific, MA, United States). Ligation was performed using final concentration of 0.06 pmol of the digested PCR amplified product and 0.02 pmol of the digested vector at 22°C for 1 h. The ligation reaction mixture was inactivated at 65°C for 10 min and used for transforming DH5α to increase the copy number of the plasmid. Subsequently it was used to transform the respective competent mutant ocular *E. coli* cells by heat shock method.

### Semi Quantitative PCR for Complement Strains

Reverse transcription-PCR was performed to validate the expression of genes in the mutants and complemented strains using primers as in Table 1. Amplification was done for 40 cycles as follows: 94°C for 5 min, 40× (94°C for 30 s, 60°C for 30 s).

### STRING Functional Analysis

The predicted functional interactions among genes *bdcR, mdtO, mhpA, mhpB, tolA*, and *ytfR* was ascertained using STRING^1^, Search Tool for the Retrieval of Interacting Genes/Proteins) analysis.

## Results

In an earlier study we had demonstrated that *E. coli* L-1216/2010 isolated from the vitreous of an endophthalmitis patient exhibited very good ability to form biofilm and in the biofilm phase it was 100 fold more resistant to several antibiotics (Ranjith et al., 2017). We had also identified several genes that were up-regulated in expression during biofilm phase (Ranjith et al., 2017). In the present study, a few of the genes up-regulated in expression during biofilm phase viz., *bdcR, mdtO, mhpA, mhpB, ryfA, tolA*, and *ytfR* (Ranjith et al., 2017) were mutated by homologous recombination (Datsenko and Wanner, 2000) to validate the function of the above genes in biofilm formation in ocular pathogenic *E. coli*. Following homologous recombination the mutants were validated by PCR and Reverse transcription-PCR. PCR confirmed the insertion of the antibiotic cassette in the target gene such that the PCR amplicon on sequencing revealed that the antibiotic cassette was intact and 36 bp of the target was present upstream and downstream of the antibiotic cassette. Further Reverse transcription-PCR also indicated that the gene is not expressed in the mutant (Supplementary Figure 1).

### Biofilm Formation and Growth of the Seven Ocular *E. coli* Mutants

**FIGURE 1:**
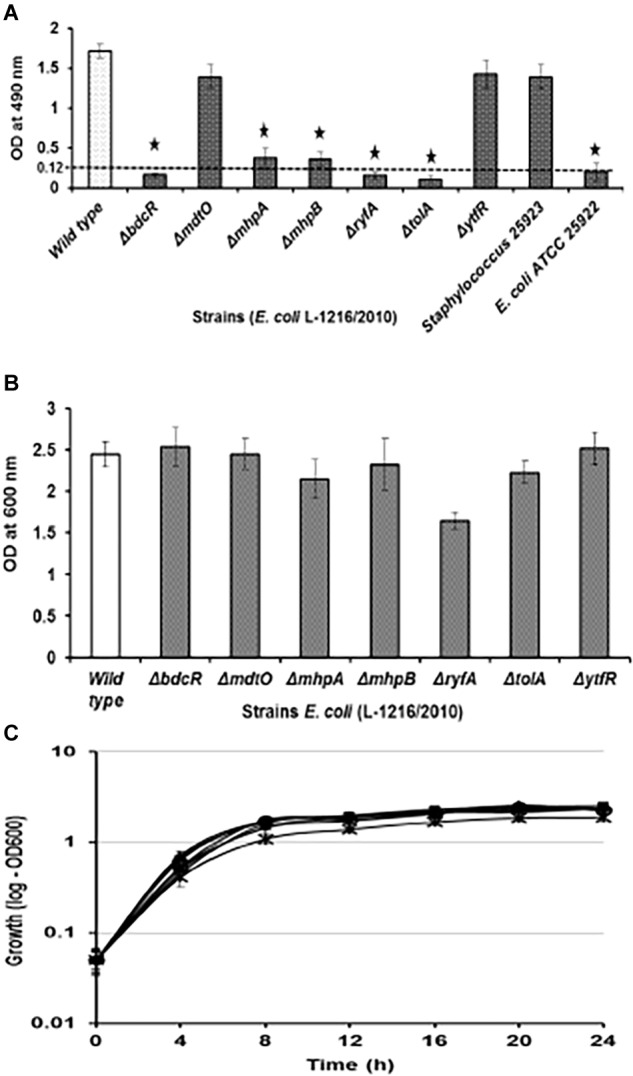
**(A)** Biofilm formation in ocular *Escherichia coli* L-1216/2010 (control) and the 7 mutants (Δ*bdcR*, Δ*mdtO*,Δ*mhpA*, Δ*mhpB, ΔryfA*,Δ*tolA*, and Δ*ytfR*) by XTT assay. *Staphylococcus* 25923 and *E. coli* ATCC 25922 served as the positive and negative controls, respectively. ^∗^ indicates significant decrease from the control (ocular *E. coli* L-1216/2010) and similar to *E. coli* ATCC 25922 in the negative control. **(B)** Growth of ocular *E. coli* L-1216/2010 (control) and the 7 mutants (Δ*bdcR*, Δ*mdtO*,Δ*mhpA*, Δ*mhpB*, Δ*ryfA*, Δ*tolA*, and Δ*ytfR*) monitored at 600 nm at 24 h. Growth in all the mutants was similar to the wild type (*p* > 0.05). **(C)** Growth curve of *E. coli* L-1216/2010 (wild type) and the 7 mutants 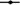
Δ*bdcR*, 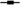 Δ*mdtO*, 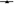 Δ*mhpA*, 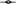
Δ*mhpB*, 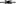
Δ*ryfA*, 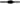
Δ*tolA*, 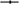
Δ*ytfR*, and 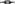
control monitored spectrophotometrically at 600 nm.

Five (Δ*bdcR*, Δ*mhpA*, Δ*mhpB*, Δ*ryfA*, and Δ*tolA*) of the seven mutants showed significant decrease in biofilm formation (*p* < 0.05) as monitored by XTT method. In all the five mutants biofilm formation by the XTT method appeared to be as in *E. coli* ATCC 25922 which served as the negative control. In contrast, the mutants Δ*mdtO* and Δ*ytfR* retained their ability to form biofilm, similar to wild type *E. coli* L-1216/2010 (*p* > 0.05) (Figure 1A). The inability to form the biofilm in the above five mutants was not dependent on its growth which was not affected compared to the control (Figure 1B). Even the growth curves of the mutants compared to *E. coli* L-1216/2010 was monitored and no significant difference was observed (Figure 1C).

### Biofilm Formation in the Seven Ocular *E. coli* Mutants by Confocal Laser Scanning Microscopy (CLSM)

**FIGURE 2:**
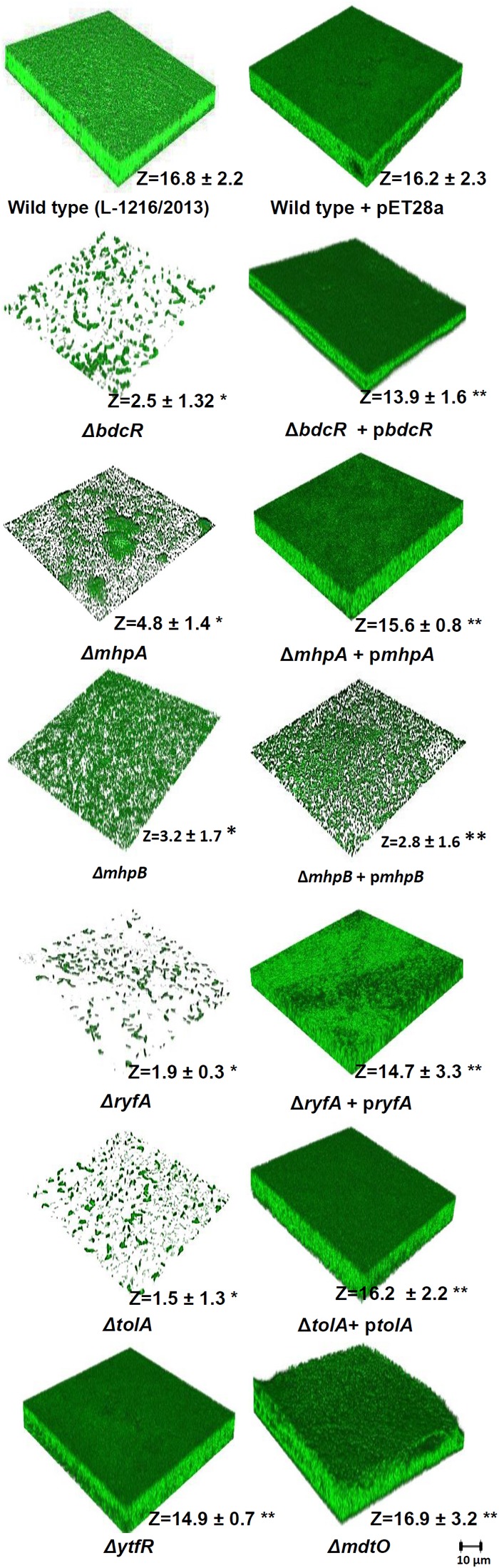
Biofilm formation in ocular *E. coli* L-1216/2010 (wild type) and the 7 mutants (Δ*bdcR*,Δ*mhpA*,Δ*mhpB*,Δ*ryfA*,Δ*tolA*,Δ*ytfR, and*Δ*mdtO*) and the mutant complemented strains of Δ*bdcR + pbdcR*, Δ*mhpA* + p*mhpA*, Δ*mhpB+* p*mhpB*, Δ*ryfA+* p*ryfA*, and Δ*tolA +*p*tolA.* The image of *E. coli* L-1216/2010 + pET28A served as control for all the complemented strains.^∗^Indicates significant decrease in thickness compared to *E. coli* L-1216/2010 (control) (*p* ≤ 0.05). ^∗∗^ indicates no significant decrease in thickness compared to *E. coli* L-1216/2010 (control) (*p* ≥ 0.05). *Z* values are expressed in μm. All images were recorded at the same magnification. The scale bar is shown in the figure.

Confocal laser scanning microscopy also confirmed that the luxuriant biofilm that formed after 72 h in *E. coli* L-1216/2010 was significantly decreased in Δ*bdcR*, Δ*mhpA*, Δ*mhpB*, Δ*ryfA*, and Δ*tolA* and was unaffected in Δ*ytfR* and Δ*mdtO* (Figure 2). The thickness of the biofilm reduced significantly from 16.8 μm in the wild type to as low as 1.5 μm in Δ*tolA*.

### Biofilm Formation of the Ocular *E. coli* Mutants and the Complemented Strains by CLSM and XTT Method

**FIGURE 3:**
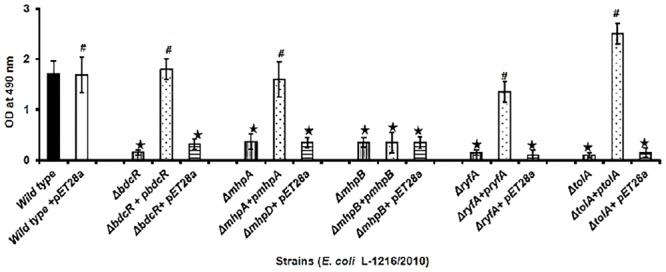
Quantification of biofilm formation by the XTT method in ocular *E. coli* L-1216/2010 (control), the 5 mutants (Δ*bdcR*, Δ*mhpA*, Δ*mhpB*, Δ*ryfA*, and Δ*tolA*) and the 5 mutant complemented strains (Δ*bdcR + pbdcR*, Δ*mhpA* + p*mhpA*, Δ*mhpB +* p*mhpB*, Δ*ryfA+*p*ryfA*, and Δ*tolA +*p*tolA*). Control and mutants complemented with pET28A served as control for the complemented strains. ^∗^ indicates significant decrease in biofilm formation compared to *E. coli* L-1216/2010 (control) (*p* ≤ 0.05). # indicates no significant decrease in biofilm formation compared to *E. coli* L-1216/2010 (control) (*p* > 0.05).

Confocal laser scanning microscopy indicated that in four of the five mutants following complementation with the respective gene (Δ*bdcR +* p*bdcR*, Δ*mhpA* + p*mhpA*, Δ*ryfA +* p*ryfA*, and Δ*tolA +* p*tolA*) biofilm formation was restored as in the control (Figure 2). Complementation also increased the thickness of the biofilm (range 13.9–16.3 μm) as observed in wild type ocular *E. coli* (16.8 + 2.2 μm) (*p* > 0.05). But, complementation did not restore the biofilm in Δ*mhpB+* p*mhpB* (Figure 2). Quantitation of the biofilm by the XTT method also confirmed that except for the mutant Δ*mhpB* complementation was efficient in restoring biofilm potential in the remaining four mutants (Figure 3).

### EPS Production in the Ocular *E. coli* L-1216/2010 and the Mutants Using Calcofluor White Staining

**FIGURE 4:**
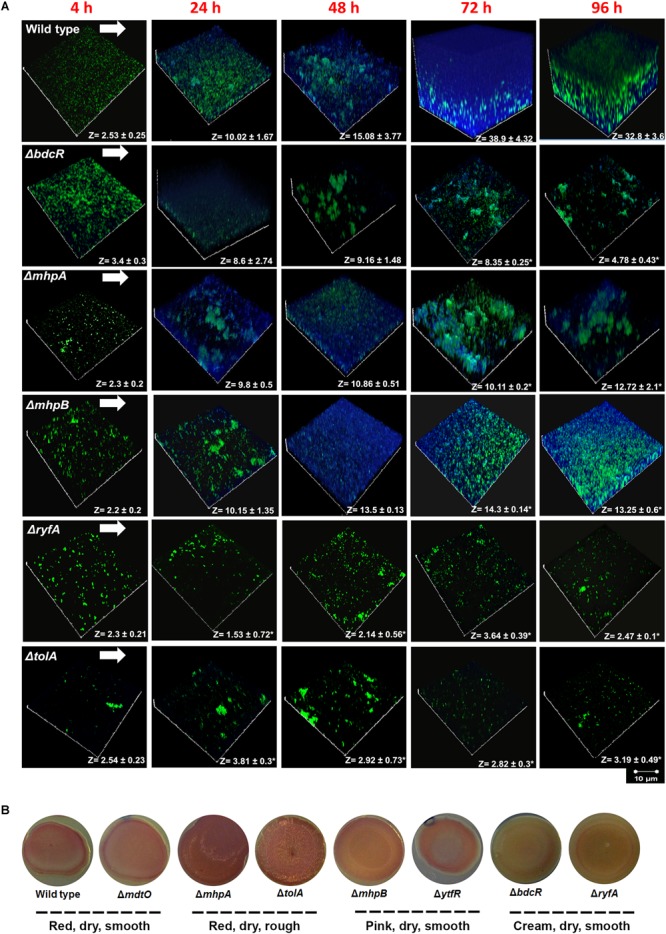
**(A)** EPS production in ocular *E. coli* L-1216/2010 (wild type l) and the 5 mutants (Δ*bdcR*, Δ*mhpA*, Δ*mhpB*, Δ*ryfA*, and Δ*tolA*). *Z* values are expressed in μm. All images were recorded at the same magnification. The scale bar is shown in the figure. ^∗^ indicates significant decrease in thickness compared to *E. coli* L-1216/2010 (wild type) (*p* ≤ 0.05). **(B)** Production of curli and cellulose nanofibers in ocular *E. coli* L-1216/2010 (wild type) and the 7 mutants (Δ*bdcR*, Δ*mdtO* Δ*mhpA*, Δ*mhpB*, Δ*ryfA*, Δ*tolA, and*Δ*ytfR*).

Using a dual staining strategy with Syto 9 for cells (green) and Calcofluor white for EPS (blue) it was observed that in the wild type cells EPS thickness increased from 2.53 μm at 4 h to 32.80 μm by 96 h. Maximum thickness was observed at 72 h. In contrast all the 5 mutants showed a significant decrease in the thickness of EPS. In 3 mutants, namely Δ*bdcR*,Δ*mhpA* and Δ*mhpB* EPS thickness increased up to 24 h but remained unchanged subsequently except Δ*bdcR* which showed substantial decrease in thickness from 72 to 96 h. The other two mutants Δ*tolA* and Δ*ryfA* did not show any significant increase in EPS between 4 to 96 h. We also observed that cells of Δ*mhpA* and Δ*mhpB* had reduced EPS production and appeared to aggregate in to clumps between 48–96 h (Figure 4A).

### Production of Curli Fimbriae and Cellulose Nanofibers by Congo Red-Binding Assay in *E. coli* L-1216/2010 and the Mutants

It was observed using Congo red-containing YESCA agar plates that out of 7 mutants, three were red in color, dry and either smooth (Δ*mdtO*) or rough (Δ*mhpA* and Δ*tolA*), two (Δ*mhpB* and Δ*ytfR*) were pink, dry and smooth, and the remaining two (Δ*bdcR* and Δ*ryfA*) were cream in color, dry and smooth. The wild type was also observed to be red, dry and smooth. These morphological features indicated that the wild type and 3 mutants that were dry and red are positive for curli fimbriae and cellulose nanofibers production. (Römling, 2005; Milanov et al., 2015), the 2 mutants that were pink and smooth were positive for cellulose nanofibers production (Zogaj et al., 2003), and the 2 mutants that were cream colored were negative for both curli fimbriae and cellulose nanofibers production.(Figure 4B).

### Swimming, Swarming, and Adhesion of Ocular *E. coli* L-1216/2010 and the Mutants

**FIGURE 5:**
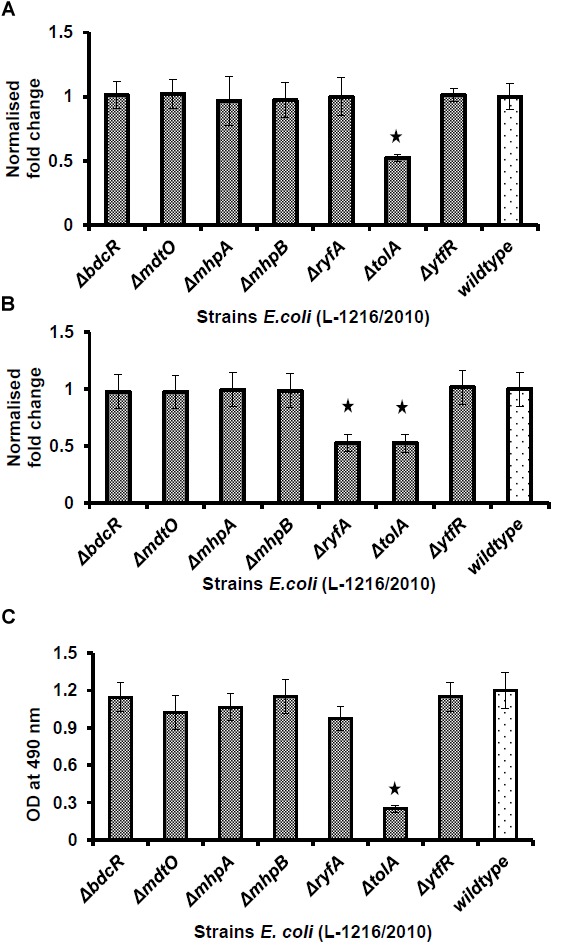
Swimming **(A)** and swarming **(B)** of ocular *E. coli* L-1216/2010 (wild type) and the 7 mutants (Δ*bdcR*, ΔmdtO, Δ*mhpA*, Δ*mhpB*, Δ*ryfA*, Δ*tolA*, and Δ*ytfR*). The diameter of the swimming and swarming zone in the wild type cells was 22 ± 3.75 mm- and 14.35 ± 2.45 mm, respectively. ^∗^ indicates significant decrease compared to *E. coli* L-1216/2010 (wild type) (*p* ≤ 0.05). **(C)** Quantification of adhesion by the syto9 staining method of ocular *E. coli* L-1216/2010 (wild type) and the 7 mutants (Δ*bdcR*, ΔmdtO, Δ*mhpA*, Δ*mhpB*, Δ*ryfA*, Δ*tolA*, and Δ*ytfR*). The adhesion of all mutants were similar to wild type cells except Δ*tolA*.

Swimming and swarming of the wild type and mutant cells was monitored overnight and the results indicated that compared to *E. coli* L-1216/2010 swimming was significantly reduced in two mutants (Δ*ryfA* and Δ*tolA, p < 0.05*) (Figure 5A). In contrast swarming was significantly reduced only in *tolA* (*p < 0.05*) (Figure 5B). Results of adhesion indicated that the number of cells attached to the substratum appeared to be similar in all except Δ*tolA* where less cells appeared to be attached (Figure 5C).

### Antibiotic Sensitivity of the Ocular *E. coli* Mutants and the Complemented Strains

The five mutants which did not form the biofilm (Δ*bdcR*, Δ*mhpA*, Δ*mhpB*, Δ*ryfA*, and Δ*tolA*) following exposure to 9 different antibiotics in the planktonic phase exhibited MICs similar to that observed for the wild type *E. coli* L-1216/2010 (Table 2). Mutant Δ*ytfR* which retained its ability to form a biofilm also had a similar MIC compared to the wild type *E. coli* in the planktonic phase. But, mutant Δ*mdtO* which retained its ability to form a biofilm showed reduced MIC to 4 out of the 9 antibiotics tested (viz., Sulfamethoxazole, Imipenem, Streptomycin and Erythromycin) (Table 2) in the planktonic phase. We also checked the MIC of the mutants Δ*ytfR* and Δ*mdtO* in response to the above four antibiotics in the their biofilm phase and observed that the MIC compared to the control was altered only in Δ*mdtO* in the biofilm phase implying that the mutation had not affected its ability to form biofilm but has altered its sensitivity to antibiotics (Table 3). Finally we also checked the antibiotic sensitivity of the complemented strains in the biofilm phase and the results indicated that the complemented strains Δ*bdcR + pbdcR*, Δ*mhpA* + p*mhpA*, and Δ*mhpB+* p*mhpB* did not differ in their sensitivity to all the 9 tested antibiotics compared to the wild type cells whereas Δ*tolA +* p*tolA* appeared to be more resistant to 2 of the 9 antibiotics (namely Gentamycin and Erythromycin) (Table 4).

**Table 2:** Antibiotic sensitivity of the ocular *E. coli* L-1216/2010 and the 7 mutants to 9 different antibiotics.

	MIC of antibiotic (μg/ml)^∗^							
Antibiotic	Wild	Δ*bdcR*	Δ*mdtO*	Δ*mhpA*	Δ*mhpB*	Δ*ryfA*	Δ*tolA*	Δ*ytfR*
Cefotaxime	16	16	16	16	16	16	16	16
Ceftazidime	12	12	12	12	12	12	12	12
Cefuroxime	28	28	28	28	28	28	28	28
Ciprofloxacin	12	12	12	12	12	12	12	12
Erythromycin	32	32	16	32	32	32	32	32
Gentamycin	12	12	12	12	12	12	12	12
Imipenem	32	32	16	32	32	32	32	32
Streptomycin	24	24	12	24	24	24	24	24
Sulfamethoxazole	24	24	16	24	24	24	24	24

*^∗^Antibiotic susceptibility test was ascertained by the drug dilution method and repeated thrice.*

**Table 3:** Antibiotic susceptibility of ocular *E. coli* L-1216/2010 and mutants Δ*ytfR* and Δ*mdtO* in the biofilm phase to 4 different antibiotics.

	MIC of antibiotic (μg/ml)^∗^					
	Wild type		Δ*mdtO*		Δ*ytfR*	
Antibiotic (μg/ml)	Planktonic	Biofilm	Planktonic	Biofilm	Planktonic	Biofilm
Erythromycin	32	512	16	64	16	512
Imipenem	32	512	16	256	32	512
Streptomycin	24	1024	12	128	24	1024
Sulfamethoxazole	24	1024	16	32	24	1024

*^∗^Antibiotic susceptibility test was ascertained by the drug dilution method and repeated thrice.*

**Table 4:** Antibiotic susceptibility of ocular *E. coli* L-1216/2010 and mutants complemented with their respective genes in the biofilm phase to 9 different antibiotics.

Antibiotic	MIC of antibiotic (μg/ml)^∗^				
	Wild	Δ*bdcR* +p*bdcR*	Δ*mhpA* + p*mhpA*	Δ*ryfA*+ p*ryfA*	Δ*tolA* + p*tolA*
Cefotaxime	256	256	256	256	256
Ceftazidime	1024	1024	1024	1024	1024
Cefuroxime	512	512	512	512	512
Ciprofloxacin	512	512	512	512	512
Erythromycin	512	512	512	512	1024
Gentamycin	512	512	512	512	> 1024
Imipenem	512	512	512	512	512
Streptomycin	1024	1024	1024	1024	1024
Sulfamethoxazole	1024	1024	1024	1024	1024

*^∗^Antibiotic susceptibility test was ascertained by the drug dilution method and repeated thrice.*

### Interaction of the Mutated Genes in *E. coli* L-1216/2010

**FIGURE 6:**
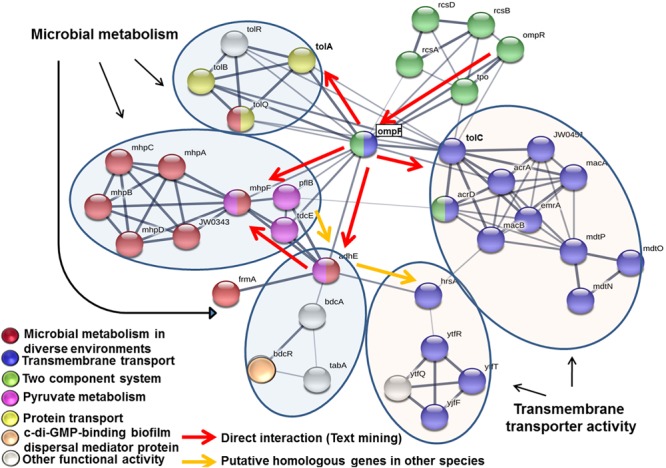
STRING network analysis of the genes *bdcR, mdtO, mhpA, mhpB, ryfA, tolA*, and *ytfR* that were mutated in ocular *E. coli* L-1216/2010. Each node in the network denotes a gene while edges between nodes indicate interactions between corresponding genes. The thickness of the edge color indicates the strength of interaction (0–1.0). In the present work, the edge confidence in the network ranged from 0.4 to 0.9. Nodes are colored based on the functional interactions. Genes involved in primary network interactions are circled.

STRING network analysis indicated that genes *bdcR, mdtO, mhpA, mhpB, tolA*, and *ytfR* were organized in to two functional arms namely microbial metabolism and transmembrane transporter activity. Genes *bdcR, mhpA, mhpB*, and *tolA* were part of the microbial metabolism arm and interacted with genes involved in pyruvate metabolism (genes *mhpF, plfB, tdcE*, and *adhE*), 3-(3-hydroxy) phenyl propionate catabolic process (genes *mhpA* and *mhpB*), acetaldehyde dehydrogenase activity (genes *mhpF* and *adhE*) and genes involved in biofilm such as c-di-GMP-binding biofilm dispersal mediator protein (*bdcR*). The remaining 2 genes *mdtO* and *ytfR* were part of the transmembrane transport activity and interacted with genes involved in transport such as protein transport genes (*tolA, tolB, tolQ*, and *tolR*) and transmembrane transport genes (*ompF, tolC, acrD, acrA*, JW0451, *macB, emrA, macA, mdtP, mdtO, mdtN, hrsA, ytfT, ytfF*, and *ytfR*) (Figure 6). The interaction between the genes was mediated by *ompR* encoding for DNA-binding transcriptional dual regulator, *ompF* encoding for outer membrane porin 1a, *adhE* and *mhpF* encoding for aldehyde dehydrogenase, *tolC* encoding for transport channel and *tolA* encoding for inner membrane protein member of Tol-Pal system. The network also included genes coding for the “two component signal transduction system” *ompR* and *rcs*A.

## Discussion

Understanding the molecular basis of biofilm formation is very crucial in identifying targets to hack biofilms so that virulent and pathogenic bacteria could be controlled. Several studies have demonstrated that in *E. coli* in the biofilm phase several hundreds of genes are differentially expressed (Schembri et al., 2003; Beloin et al., 2004; Ren et al., 2004; Domka et al., 2006; Hancock and Klemm, 2007; Ranjith et al., 2017). In a recent study we demonstrated that in ocular *E. coli* L-1216/2010 also several genes were differentially expressed in the biofilm phase (Ranjith et al., 2017). Further, ocular *E. coli* could be differentiated from *E. coli* K12, a laboratory non-pathogenic strain of *E. coli*, with respect to expression of genes involved in cell cycle control, mitosis and meiosis, cell motility, intracellular trafficking, secretion, vesicular transport and defense mechanisms (Ranjith et al., 2017) implying that ocular *E. coli* is probably more like a pathogen. In fact this may indeed be so because the ocular *E. coli* L-1216/2010 and ABU *E. coli* (Ranjith et al., 2017) a pathogenic strain, were similar with respect to differential regulation of genes involved in intracellular trafficking, secretion and vesicular transport, defense mechanisms and replication, recombination, and repair which are normally associated with pathogenic bacteria. In the present study the question addressed is whether ocular pathogen are similar to other pathogens with respect to genes involved in biofilm formation and associated drug resistance.

Over the years it has been reasonably well established that genes involved in motility (Wood et al., 2006), that facilitate attachment to substratum (Prigent-Combaret et al., 2000; Ren et al., 2005), that code for production of extracellular polymers and adhesive factors (Danese et al., 2000; Schembri et al., 2004), that help in formation and maturation of micro-colonies and in cell signaling (quorum sensing) (Reisner et al., 2003) are associated with biofilm formation. A few of the genes such as *tolA, ryfA* (Whiteley et al., 2001), *ymdB* (Kim et al., 2013), *csrA* (Jackson et al., 2002), *spoT* and *relA* (de la Fuente-Nunez et al., 2014), and *ydgG* (Herzberg et al., 2006) have been shown to be required for biofilm formation by specific knock out of the gene (s) either in *P. aeruginosa* or in *E. coli* (Vianney et al., 2005). In the present study seven gene knock outs were generated in ocular *E. coli* L-1216/2010 to ascertain the role of these genes (*bdcR, mdtO, mhpA, mhpB, tolA, ryfA*, and *ytfR*) in biofilm formation. Earlier studies had indicated that knockout mutants of two genes *ryfA* and *tolA* had lost the potential to form biofilm (Whiteley et al., 2001; Vianney et al., 2005). In this study we confirm that in ocular *E. coli, ryfA* which codes for a small RNA (Bak et al., 2015) following deletion exhibits a decrease in biofilm development probably due to the observed decrease in EPS production, cellulose nanofibers and curli production. The mutant also exhibited significant decrease in swarming. Earlier studies had indicated that curli are important for biofilm formation by facilitating initial attachment to a surface and cell-to-cell cohesion (Pratt and Kolter, 1999). In an earlier study it was demonstrated that in *P. aeruginosa, tolA* gene was activated in biofilms (Whiteley et al., 2001) and mutation of the gene in *E. coli* MG1655 and two clinical strains of *E. coli* PHL881 and PHL885 led to a decrease in biofilm formation implying the importance of *tolA* in biofilm formation (Vianney et al., 2005). They also attributed the decrease in biofilm formation to the observation that in the *tol* mutants the adherence of cells to the substratum was lowered. The present study confirms both the observations that in Δ*tolA* mutants of ocular *E. coli* biofilm formation is decreased and it could be attributed to a decrease in adhesion of cells, swimming, swarming and production of EPS. Interestingly it was observed that in the mutant, both curli and cellulose nanofibers production was not affected implying that curli is not mandatory for biofilm formation. The reports on the requirement of curli for biofilm formation are contradictory with a few indicating that curli and cellulose nanofibers are not required for biofilm formation (Niba et al., 2007; Cegelski et al., 2009) unlike the report of Arita-Morioka et al. (2015). It also appears that since *tolA* interacts with other *tol* genes (*tolB, tolQ*, and *tolR*), with *ompF* involved in bacteriocin (*tolA, tolB, tolQ*, and *tolR*) and drug transport (*acrD, acrA, JW0451, macB, emrA, mdtP, mdtO*, and *mdtN*) and *tolC* which regulates genes involved in transmembrane transport activity (Figure 6) it may contribute to drug resistance. But mutants did not show any change in susceptibility to 9 antibiotics tested implying that *tolA* may not be directly involved in drug resistance.

We had earlier shown that many genes encoding dehydrogenases (*mhpA, mhpB, mhpF, yiaY, yajO/ydbK*, and *yjjN*) were up regulated during biofilm formation in ocular *E. coli* (Ranjith et al., 2017) implying their likely role in biofilm formation. But none of these genes were demonstrated to be required for biofilm formation. In this study we demonstrate that biofilm formation was decreased substantially in separate knockout mutants of *mhpA* and *mhpB* implying that these two genes are required for biofilm formation. *mhpA* and *mhpB* are specifically involved in 3-(3-hydroxy) phenyl propionate catabolic process and thus it is likely that these two genes may be associated with a metabolic function. In fact, String network analysis also indicated a close interaction between *mhpA* and *mhpB* with other microbial metabolic genes such as genes involved in pyruvate metabolism (genes *mhp*F, *plf*B, *tdc*E, and *adh*E) and genes coding for acetaldehyde dehydrogenases (genes *mhp*F and *adh*E) and could thus be involved in microbial metabolism, drug metabolism and support drug resistance a phenomenon associated with biofilm formation. But their role in drug resistance is unlikely since it was observed that the mutants susceptibility to the nine antibiotics tested remained unchanged. When we investigated the phenotypic characteristics it was observed that both the mutants (Δ*mhpA* and Δ*mhpB*) ability to swim, swarm and attachment remained unaffected. In addition it was observed that curli and nanofiber production was not affected in *mphA* but in contrast, Δ*mhpB* only curli production was affected.

In ocular *E. coli* it was demonstrated that the genes *bdcA* and *bdcR* were upregulated more than 20 fold in the biofilm phase implying their need for biofilm formation (Ranjith et al., 2017). It has been demonstrated that *bdcA* deletion reduces biofilm dispersal by decreasing motility and thus does not affect biofilm formation (Ma et al., 2011). In accordance with the above observation in the present study it was observed that knockout mutants of *bdcR*, a negative regulator of *bdcA*, and showed reduced biofilm formation. But the mutants ability to swim, swarm and attach was not altered but EPS, curli and cellulose nanofiber production was significantly decreased. Thus it appears that *bdcR* is required for biofilm formation in ocular *E. coli*. It was also observed that unlike the wild type cells, knockout mutants of *mhpA, mhpB*, and *bdcR* showed clumping of cells at some time points during the biofilm phase.

Genes coding for ABC transporter ATP-binding protein and belonging to the ATP-binding cassette (ABC) transporter superfamily have been implicated in biofilm formation. But their involvement in biofilm formation appears to vary from organism to organism. For instance in *Rhizobium leguminosarum* mutation in the ATP-binding protein of an uncharacterized ABC transporter operon significantly reduced the number of viable cells and simultaneously inhibited biofilm formation suggesting that a functional transporter is essential for normal biofilm formation (Vanderlinde et al., 2010). In contrast, (Zhu et al., 2008; Ceruso et al., 2014) observed that in *Listeria monocytogenes*, inactivation of the putative ABC transporter or the permease component, caused enhanced biofilm-formation compared to the wild type, indicating that *LMOf 2365_1875* negatively regulates biofilm formation. At the same time (Ceruso et al., 2014) also observed that *LMOf 2365_1877* another mutant in the ABC transporter gene did not have any effect on biofilm formation. In the present study we observed that deletion of *ytfR* (a gene that codes for ABC transporter ATP-binding protein) in ocular *E. coli* did not alter its biofilm formation potential as observed in the above studies and also did not affect the motility, initial attachment and celluose nanofiber production but curli production was affected. It is possible that the function of *ytfR* is compensated by other genes of the ABC transporter superfamily. In fact, String analysis does indicate that *ytfR* closely interacts with other members of the ABC transporter superfamily like *ytfT, yjfF, ytfQ*, and *hrs*A which may functionally compensate for its deletion. Recently it was demonstrated by Pinweha et al. (2018) that in *Burkholderia pseudomallei*, ABC transporter mutant *bpsl1039-1040* showed reduced biofilm formation as compared with the wild-type strain (*P* = 0.027) when cultured in LB medium supplemented with nitrate under anaerobic growth conditions. But this reduction in biofilm formation was not noticeable under aerobic conditions. Thus overall it would appear that ABC transporter gene function with respect to biofilm formation varies not only from organism to organism but also under the physiological condition it is tested.

Among the various genes that were upregulated in ocular *E. coli* we also noticed that genes involved in multidrug resistance like *mdtO* (synonym *yjcQ*) were also up regulated in their biofilm phase in ocular *E. coli*. This is of interest since biofilm formation is related to resistance to antibiotics (Kaplan, 2011) but the correlation appears to be inconsistent and the results are conflicting. For instance in *P. aeruginosa* and *Acinetobacter baumannii* biofilm production was significantly higher in multidrug resistant (MDR) isolates (Abidi et al., 2013; Gurung et al., 2013; Qi et al., 2016). But it was not so in MDR *S. aureus* (Eyoh et al., 2014). In this study we observed that in *mdtO* deletion mutant of *ocular E. coli* formation of biofilm was unaltered and all the attributes of a biofilm forming bacterium such as swimming, swarming, attachment and production of curli, and cellulose nanofibers were unaltered. But the susceptibility of the *mdtO* deletion mutant to Sulfamethoxazole, Imipenem, Erythromycin, and Streptomycin was increased as judged by the lower MICs. Thus the observations emphasize a role for the gene exclusively in drug resistance.

In the current study, out of the five deletion mutants (Δ*bdcR*, Δ*mhpA*, Δ*mhpB*, Δ*tolA*, and Δ*ryfA*) complementation was successful in all the mutants except Δ*mhpB* in which the biofilm formation potential was not restored. Further the plasmid used for complementation had no effect on the growth. In two mutants of ABC transporter in *Rhizobium leguminosarum* attempts to restore wild-type phenotype of biofilm formation by complementation was also not successful (Vanderlinde et al., 2010) and was attributed to a dominant-negative effect as observed earlier for other ABC transporters (Bliss et al., 1996; Miyamoto et al., 2002; Eyoh et al., 2014). In the present case it needs to be established.

The STRING network analysis of the deleted genes in *E. coli* appeared to be involved in two functional arms namely “microbial metabolism” and “transmembrane transporter activity.” Genes *mhp*A, *mhp*B, *bdc*R, and *tol*A constituted the metabolism arm and deletion of these genes decreased biofilm formation but their susceptibility to antibiotics in the planktonic phase was unaltered. It appears that these genes involved in microbial metabolism are essential for biofilm formation and supports a previous study that acetate metabolism is related to the formation of biofilms in *E. coli* (Pruss et al., 2010). The four genes *bdcR, mhpA, mhpB*, and *tolA* of the metabolic arm also interacted with other metabolic genes of pyruvate metabolism, 3-(3-hydroxy) phenyl propionate catabolic process and acetaldehyde dehydrogenase activity regulated either directly or indirectly by their interaction with *ompF* (outer membrane porin). In contrast, *E. coli* cells mutated for genes *ytfR* and *mdtO* were implicated in “transmembrane transporter activity” and deletion of these genes did not alter their ability to produce biofilm as in wild type cells. But, one of the mutants Δ*mdtO* showed more sensitivity to Sulfamethoxazole, Imipenem, Streptomycin, and Erythromycin both in the planktonic and biofilm phase. Thus these genes appear to be more related to drug resistance. *ompF* also regulated this arm by indirectly interacting with *mdtO* through *tolC* and with *ytfR* through *adhE.* This arm also included genes involved in drug transport (*ompF, acrD, acrA, JW0451, macB, emrA, mdtP, mdtO*, and *mdtN*) and sugar transport genes (*hrsA, ytfT, ytfF*, and *ytfR*). It is also observed that genes coding for the “two component signal transduction system” *ompR* and *rcsA* (Pruss, 2017) were central to regulating the two arms through *ompF.* Thus it is possible that genes of the “microbial metabolism” arm are required for biofilm formation whereas genes of the “transmembrane transporter activity” are required for drug resistance and not essential for biofilm formation.

## Conclusion

In the present study we mutated a total of seven genes in ocular *E. coli* L-1216/2010 out of which in five mutants (Δ*bdcR*, Δ*mhpA*, Δ*mhpB*, Δ*ryfA*, and Δ*tolA*) biofilm formation was inhibited. In three of the mutants (Δ*bdcR*, Δ*mhpA*, and Δ*mhpB)* swimming, swarming and adhesion were not affected. In Δ*tolA* swimming, swarming and adhesion were affected. where as in Δ*ryfA* only swarming was affected. In addition, all the five mutants exhibited decrease in EPS production and in two of the mutants (Δ*bdcR* and Δ*ryfA*) curli and cellulose nanofibers production was also inhibited. Thus the only feature that appears to be shared by all the five mutants is their inability to produce EPS. In the remaining two mutants (Δ*mdtO* and Δ*ytfR*) biofilm formation was unaltered. Out of these five genes which are demonstrated to be required for biofilm formation *ryfA* and *tolA* were earlier demonstrated to be required for biofilm formation (Vianney et al., 2005; Bak et al., 2015). Thus we report for the first time that genes *bdcR*, Δ*mhpA*, and Δ*mhpB* are required for biofilm formation in ocular *E coli*. We also report that gene *mdtO*, a transmembrane protein involved in the multi-drug transport of sulphanilamide drugs in planktonic phase, when mutated did not affect the biofilm formation in ocular *E. coli* L-1216/2010 but the mutant was more sensitive to Sulfamethoxazole, Imipenem, Erythromycin, and Streptomycin both in the planktonic and biofilm phase. Thus mitigation of biofilm formation to overcome antibiotic resistance could be achieved by targeting the genes *bdcR, mhpA, mhpB, tolA*, and *ryfA*.

## Author Contributions

KR performed the experiments and participated in the data analysis. JR and JP helped in generating knockout and complementation and provided the logistic support. KA helped in string analysis. SShi participated in manuscript writing and editing. SSha conceived the project, executed the project, and helped in manuscript writing and finalization. All authors read and approved the final manuscript.

## Conflict of Interest Statement

The authors declare that the researchwas conducted in the absence of any commercial or financial relationships that could be construed as a potential conflict of interest.

## Footnotes

1 http://string-db.org/
