# Supplementary material for: Gene Targets in Ocular Pathogenic Escherichia coli for Mitigation of Biofilm Formation to Overcome Antibiotic Resistance
Source: Front Microbiol. 2019 Jun 21;10:1308. doi: 10.3389/fmicb.2019.01308 (PMC6598151; doi:10.3389/fmicb.2019.01308)

**Supplementary Figure 1.** Expression of genes in ocular *E. coli* L-1216/2010 and the 7 mutants (Δ*bdcR*, Δ*mdtO*,Δ*mhpA*, Δ*mhpB*, Δ*ryfA*, Δ*tolA* and Δ*ytfR*) and the 5 complemented strains Δ*bdcR + pbdcR*, Δ*mhpA* + p*mhpA*, Δ*mhpB+* p*mhpB*, Δ*ryfA+*p*ryfA* and Δ*tolA +*p*tolA.* Ocular *E. coli* L-1216/2010 plus pET28a the expression vector served as a control for the complemented strains*.*pgene represents the complimenting gene.


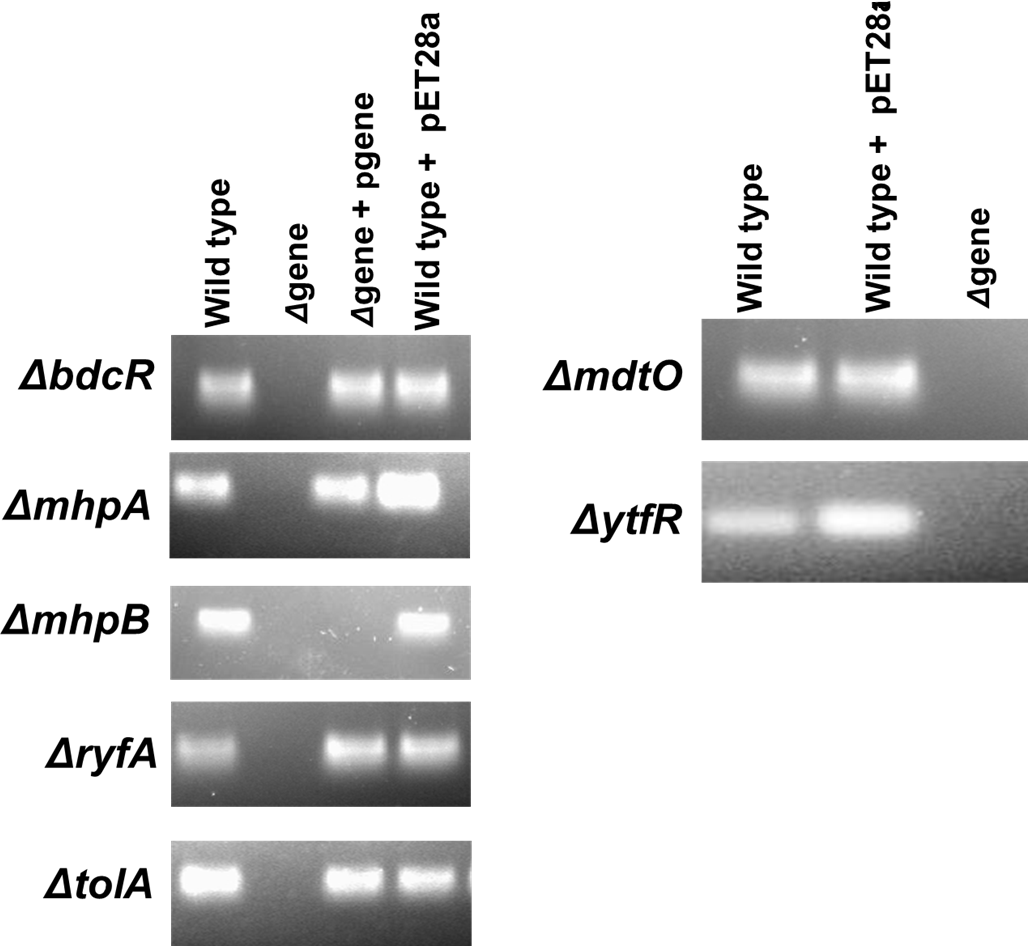

Supplement: Supplementary file 1 [file Data_Sheet_1.docx]
